# Pyrrole-Based Conjugated Microporous Polymers as Efficient Heterogeneous Catalysts for Knoevenagel Condensation

**DOI:** 10.3389/fchem.2021.687183

**Published:** 2021-05-10

**Authors:** Ruidong Gao, Guang Zhang, Fanli Lu, Long Chen, Yang Li

**Affiliations:** ^1^ Department of Chemistry, Tianjin Key Laboratory of Molecular Optoelectronic Science, Tianjin University, Tianjin, China; ^2^ College of Physics and Optoelectronic Engineering, Shenzhen University, Shenzhen, China

**Keywords:** heterogeneous catalysis, pyrrole, knoevenagel condensation, conjugated microporous polymers, photocatalysis

## Abstract

Conjugated microporous polymers (CMPs) with robust architectures, facilely tunable pore sizes and large specific surface areas have emerged as an important class of porous materials due to their demonstrated prospects in various fields, e.g. gas storage/separation and heterogeneous catalysis. Herein, two new pyrrole-based CMPs with large specific surface areas and good stabilities were successfully prepared by one-step oxidative self-polycondensation of 1,2,4,5-tetra (pyrrol-2-ly)benzene or 1,3,5-tri (pyrrol-2-ly)benzene, respectively. Interestingly, both CMPs showed very high catalytic activity toward Knoevenagel condensation reaction, which was attributed to the inherent pore channels, high specific surface areas and abundant nitrogen sites within CMPs. Additionally, both CMPs displayed excellent recyclability with negligible degradation after 10 cycles. This work provides new possibilities into designing novel nitrogen-rich high-performance heterogeneous catalysts.

## Introduction

Porous materials play significantly important roles in many fields of science and technology and have resurged with great popularity within last two decades. This, in part, is due to the emerging several kinds of unprecedented architectures with intriguing properties, e.g. metal-organic frameworks (Jiao et al., 2019), covalent organic frameworks (Cote et al., 2005) and conjugated microporous polymers (CMPs) (Cooper, 2009). CMPs are conjugated 2-dimensional or 3-dimensional polymers in contrast to many other porous materials and therefore are rigid and shape-persistent. Different from COFs, CMPs (Yue et al., 2020; Xu et al., 2021) are synthesized under kinetic control and are generally amorphous; thereby CMPs are more stable and obtained easier than COFs due to the much more kinds of reactions available for construction of CMPs, such as Sonogashira-Hagihara coupling reaction (Jiang et al., 2007) and Buchwald-Hartwig coupling reaction (Liao et al., 2018). Besides extended conjugation and high flexibility in structural design, CMPs also bear the merits of permanent porosity and tunable pore sizes. These characteristics of CMPs confer them with diverse potential applications (Lee and Cooper, 2020). For example, their π-conjugation has endowed CMPs with abundant electronic properties which have been employed to develop photocatalysts (Zhao et al., 2018) and light harvesting materials (Chen et al., 2010a). In addition, CMPs also exhibit promising prospects in heterogeneous catalysis (Chen et al., 2010a; Jiang et al., 2011), gas adsorption (Dawson et al., 2011; Lu et al., 2012), light emission (Xu et al., 2011), chemical sensors (Liu et al., 2012), energy storage (Kou et al., 2011; Xu et al., 2014; Yue et al., 2020), and biosensing (Gu et al., 2014; Ding and Han, 2015; Tan et al., 2015; Rengaraj et al., 2016).

Catalyst is an indispensable part of organic synthesis. Unfortunately, thus far, many catalysts used in industry are still non recyclable. For a sustainable future, developing reusable heterogeneous catalysts is regarded as an environmentally benign approach due to their easy separation and cleaning processes after reactions (Sartori et al., 2004). In this regard, porous materials, e.g. MOFs (Huang et al., 2020), COFs (Zhao et al., 2020) and CMPs (Tantisriyanurak et al., 2020; Xu et al., 2021) have been demonstrated as promising platforms to develop recyclable heterogeneous catalysts in part due to their large specific surface areas which could accommodate abundant guest molecules and afford many nanoreactors. In particular, researchers have been actively studying CMP-based heterogeneous catalysts over the last decade due to their insolubility in common organic solvents, high stability, inherent porosity and tailor-made functionality through facile structural design. For example, CMPs could serve as the nanoporous scaffolds for metals support to mediate catalysis (Schmidt et al., 2009; Chan-Thaw et al., 2010; Hasell et al., 2010; Gu et al., 2014). In addition, CMPs could also function as catalysts for various chemical transformations, e.g. CO_2_ reduction reaction (Hou et al., 2020), water splitting for hydrogen production (Zhao et al., 2018), erobic oxidations (Jiang et al., 2020), a-alkylation of aldehydes (Luo et al., 2015), Knoevenagel condensation (Feng et al., 2017) and singlet oxygen generation (Zhang et al., 2013). However, the cost-effective CMP based heterogeneous catalysts with excellent catalytic performances is still very rare. Thus, the development of CMP based heterogeneous catalysts is highly desired and continuously attracting growing research interests.

Pyrrole is a widely used monomer for constructing various functional materials. For example, polypyrrole represents as one of the state-of-the-art conductive polymer (Vernitskaya and Efimov, 1997), and three-dimensional polypyrroles were developed due to their enhanced performances in supercapacitors, sensors, etc. compared with linear polypyrroles. Porphyrin as a 4-fold pyrrole analog serves as a versatile monomer to construct all kinds of architectures like porphyrin-based belts (Minotto et al., 2021), polymers (Day et al., 2015), MOFs (Zhang et al., 2015), COFs (Hao et al., 2019) and CMPs (Chen et al., 2010b) for diverse applications. On account of the many functions and broad prospects of pyrrole-based materials, it is interesting to develop new kind of pyrrole-based architectures and explore their properties and applications. In this respect, even though several porphyrin-based CMPs have been reported (Chen et al., 2010b; Modak et al., 2013; Liu et al., 2014; Xu et al., 2019; Zhu et al., 2020), to the best of our knowledge, pyrrole-based CMPs are very rare (Lee and Cooper, 2020).

**SCHEME 1:**
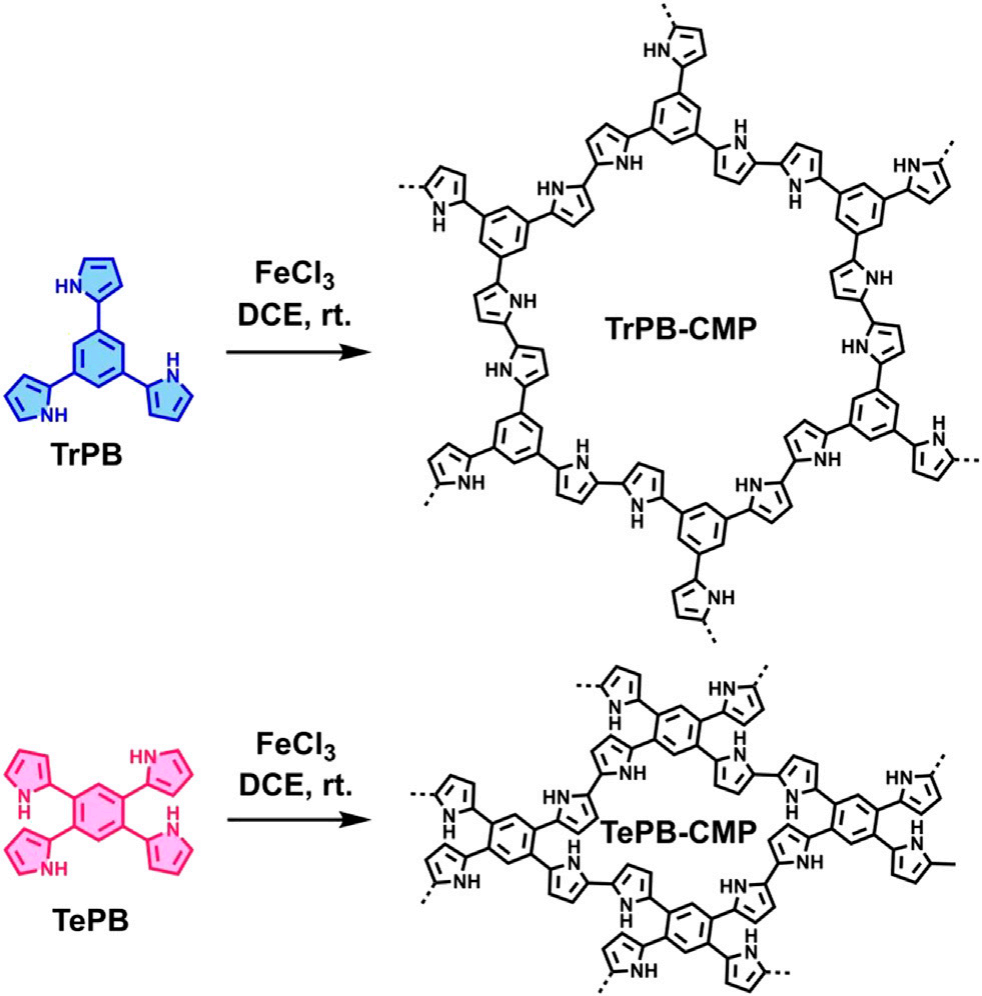
Synthetic routes for TrPB-CMP and TePB-CMP.

Herein, we designed and synthesized two new pyrrole-based CMPs (TrPB-CMP and TePB-CMP) through a simple FeCl_3_-oxidized self-condensation of multitopic pyrrole monomers (Scheme 1). We further characterized the structures and explored the properties of both CMPs with different techniques and then evaluated their catalytic performances toward Knoevenagel condensation reaction. Remarkably, both CMPs exhibit excellent catalytic activity and show superior recyclability.

## Experimental

### Synthesis of Pyrrole-Based Monomers and CMPs

The corresponding pyrrole-based monomers, i.e. 1,3,5-tri (pyrrol-2-ly) benzene and 1,2,4,5-tetra (pyrrol-2-ly) benzene (Xue et al., 2019) (Scheme 1) were readily prepared by one-step Suzuki coupling reaction between 1-(tert-butoxycarbonyl)-pyrrole-2-boronic acid and 1,3,5-tribromobenzene or 1,2,4,5-tetra-bromobenzene respectively (supporting information). Subsequently, both CMPs were synthesized by oxidative self-polymerization within chloroform at room temperature (Supplementary, ESI).

## Results and Discussion

### Structural Characterizations

The structures of both CMPs were characterized by Fourier transform infrared (FT-IR) and solid-state ^13^C cross-polarization magic angle spinning nuclear magnetic resonance (CP-MAS NMR) spectroscopies. As for FT-IR spectra of both pyrrole-based CMPs (Supplementary Figures S5, S6, ESI), the bands between 3450 and 3200 cm^−1^ correspond to the stretching vibrations of amino moieties (-NH-) originated from pyrroles (Soliman et al., 2007; Mohamed et al., 2008; Karabacak and Cinar, 2012). In addition, the bands at 1250 cm^−1^ are attributed to the -C-N- stretching vibrations (Zhang et al., 2004; Cai et al., 2011). The peaks at 1408 cm^−1^ for both CMPs are assignable to the stretching vibrations of -C=C- in the aromatic rings (Svatos and Attygalle, 1997; Samran et al., 2004). ^13^CP-MAS NMR spectra display broad signals between 100 and 140 ppm, which are attributed to the carbon signals from pyrrole and benzene rings (Supplementary Figures S13, S14, ESI) and the positions of these peaks are also in accordance with those of the monomers.

### Properties of CMPs

The crystallinities of these polymers were determined by powder X-ray diffraction (PXRD) measurements (Supplementary Figure S10, ESI). Both materials show merely a broad diffraction band between 15° and 35°, which suggests both TrPB-CMP and TePB-CMP are amorphous in nature.

**FIGURE 1:**
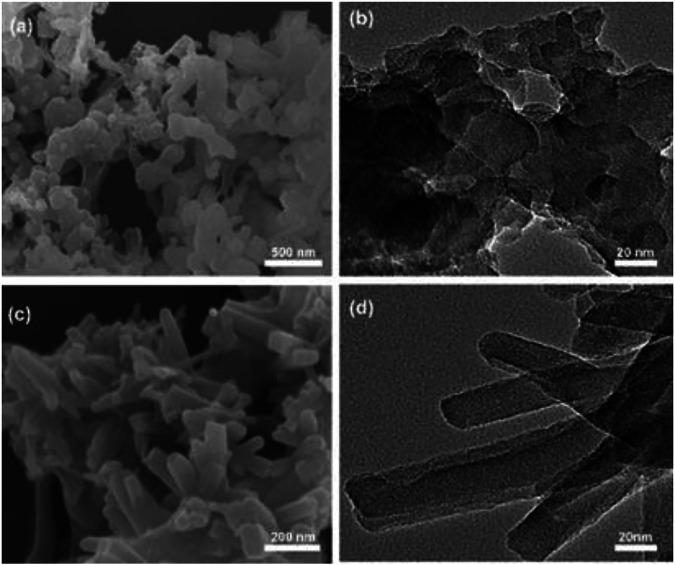
**(A)** SEM image of TrPB-CMP, **(B)** TEM image of TrPB-CMP, **(C)** SEM image of TePB-CMP and **(D)** TEM image of TePB-CMP.

To gauge the thermal stabilities of the CMPs, thermal gravimetric analysis (TGA) under nitrogen atmosphere were carried out for both materials. The curves indicate that the weights remain 97% for TrPB-CMP at 221°C and TePB-CMP at 234°C respectively (Supplementary Figure S11, ESI), further increasing the temperature renders rapid weight losses with 69% of the initial weights at 800°C, which corresponds to the degradation of the materials. To probe the photophysical properties of the CMPs, solid state diffuse reflectance UV-vis spectra of the TrPB-CMP and TePB-CMP were measured (Supplementary Figure S12, ESI). Both TrPB-CMP and TePB-CMP exhibited broad absorption band centered at 572 and 526 nm, respectively, which is assignable to the π-π* transitions of pyrrole-based conjugated networks within CMPs. Remarkably, the absorption edges of both CMPs extend to the short-wavelength infrared region (up to 2000 nm). Moreover, the morphologies of both microporous polymers were investigated by field-emission scanning electron microscopy (FE-SEM) and transmission electron microscopy (TEM). SEM images reveal TrPB-CMP consists of submicrometer-sized spheres while TePB-CMP is composed of submicrometer-sized flakes (Figure 1). In addition, TEM images show that the pore does not produce a specific texture which verify the amorphous nature of both CMPs.

**FIGURE 2:**
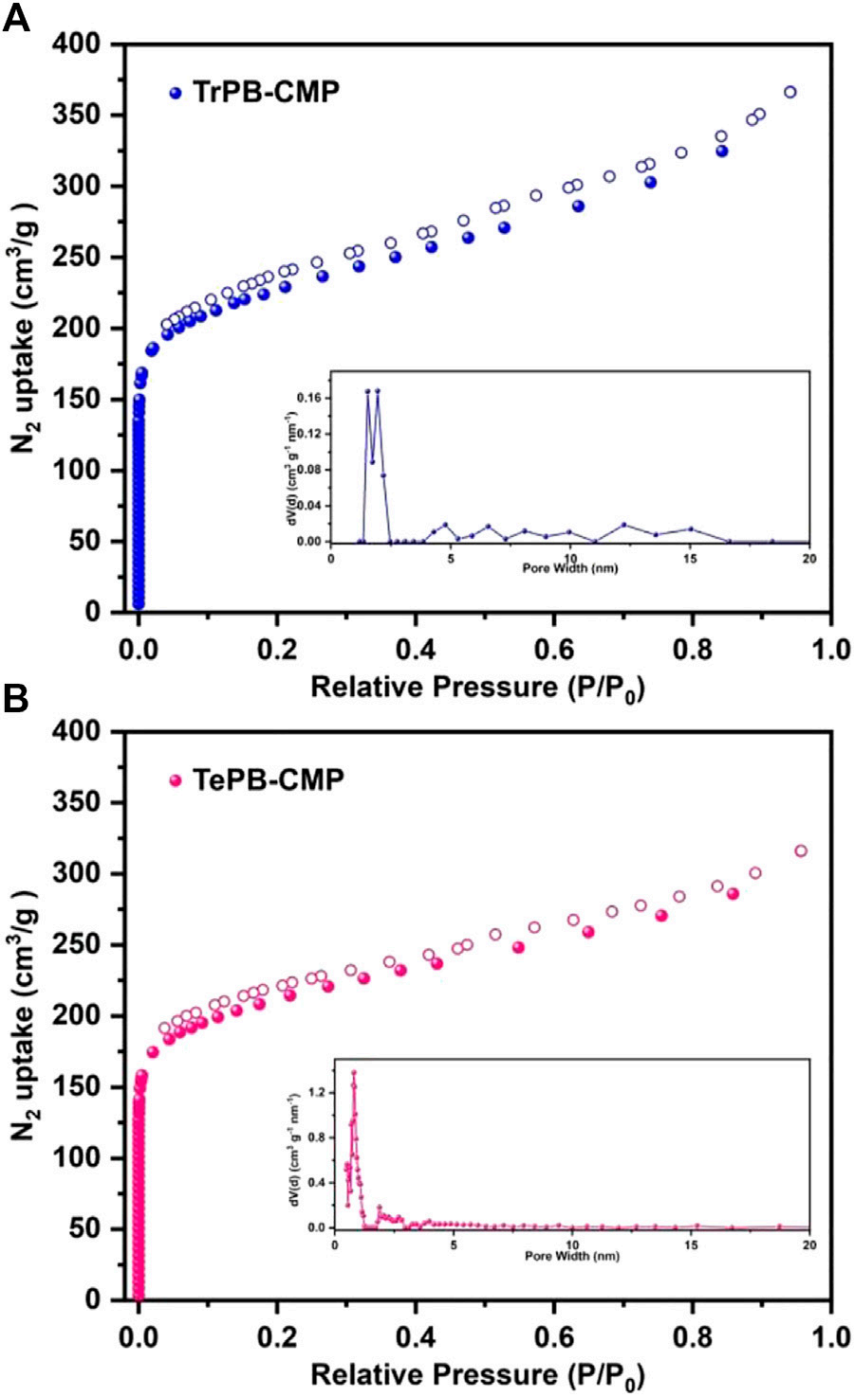
Nitrogen adsorption and desorption isotherms of TrPB-CMP **(A)** and TePB-CMP **(B)** and simulated pore size distributions of CMPs (insets) at 77 K.

The porosities of CMPs were evaluated by nitrogen (N_2_) sorption measurements. As shown in Figure 2, the nitrogen adsorption rate is extremely fast in the low relative pressure range, which indicates CMPs possess micropores. The hysteresis loop appeared in the middle pressure range of N_2_ adsorption curves indicates the existence of mesopores in CMPs (Thommes et al., 2015). The Brunauer-Emmett-Teller (BET) specific surface areas of TrPB-CMP and TePB-CMP were calculated as 810 and 800 m^2^g^−1^ respectively. The pore size distributions (PSDs) of CMPs were computed based on the adsorption branch by nonlocal density functional theory (NLDFT) method, which showed the average pore sizes of TrPB-CMP and TePB-CMP were around 1.53 and 0.80 nm respectively. Interestingly, the pore size of TrPB-CMP obtained by theoretically modeling one hexagonal segment (Supplementary Figure S18, ESI) was around 1.51 nm, which was in good consistence with the experimental result. While the pore size of TePB-CMP obtained by theoretically modeling one hexagonal segment (Supplementary Figure S19, ESI) was around 0.7 nm, which was also close to the experimental result.

### Catalytic Performances Toward Knoevenagel Condensation

Considering the presence of weakly basic pyrrole moieties within both CMPs, the CMPs might be used as heterogeneous catalysts for base-catalyzed reactions which are extremely important in catalyzing the synthesis of various small molecules for chemical and pharmaceutical industries (Perryman et al., 2013; Volchkov and Lee, 2013; Denmark et al., 2014). In this respect, base-catalyzed Knoevenagel condensation was selected as the model reaction to evaluate the catalytic activity of both CMPs. Knoevenagel condensation as a well-known and powerful reaction to formulate -C=C- bonds, exhibits broad applications in producing natural products, fine chemicals and pharmaceuticals (Knoevenagel and Dtsch, 1898; Khare et al., 2019). Recently, some representative exploratory researches on Knoevenagel condensation with porous materials as the catalysts were reported including benzimidazole-based porous organic polymers (Wang et al., 2015), 3D imine-linked COF (Fang et al., 2014), and porphyrin-based porous polymer (Modak et al., 2013).

Various reaction substrates were used to test the catalytic activities of the CMP catalysts under classical reaction conditions (Wang et al., 2015; Taher et al., 2020). In addition, the reaction temperature, solvent and reaction temperature were investigated in details to find the best conditions for the reaction (Supplementary Table S2, ESI). The yields of the substrates in the Knoevenagel condensation reaction were summarized in Table 1. As displayed in Table 1, remarkably, the reactions were completed after 1 h and the yields for all substrates under the catalysis of CMPs were quite high, which was much higher than that without addition of CMPs (44%) (Supplementary Table S3, entry 1, ESI). As for benzaldehydes with strong electron-withdrawing substituents in the para-position, the catalytic efficiency of both TrPB-CMP and TePB-CMP are basically the same with nearly quantitative conversions (entries 4, 5). The catalytic effects of both CMPs proved to be obviously different when the electron-withdrawing strength of the *para*-substituent on benzaldehyde was weakened (entries 2 and 3). It suggests TrPB-CMP renders higher conversions than TePB-CMP for the benzaldehyde substrates, which is probably due to more adequate interactions between the substrates and the basic sites within the pores of TrPB-CMP than those of TePB-CMP rendered by the bigger pore size of TrPB-CMP (entries 1 and 2). Moreover, for larger size molecules, there is a significant difference in catalytic efficiency, probably because the steric hindrance of the larger substrate molecules is not conducive to entering the micropores (entry 7). When using benzaldehyde substrates with electron-donating substituents, the catalytic yields of both TrPB-CMP and TePB-CMP were lower (entries 8 and 9). In addition, compared with the results reported in the previous literatures, the reaction conditions of the current work have advantages over others, e.g., metal-free catalysis and shorter reaction time (Supplementary Table S4, ESI).

**TABLE 1:** Catalytic activities of TrPB-CMP or TePB-CMP toward Knoevenagel condensation with different aromatic aldehyde substrates. 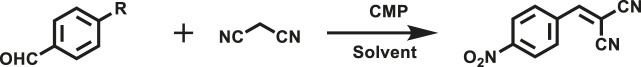

Entry	R	Substrates	Product	Yield (%)^a^	
				TrPB-CMP	TePB-CMP
1	H	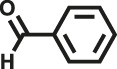	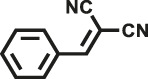	74	66
2	Br	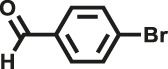	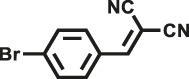	79	74
3	OH	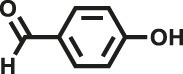	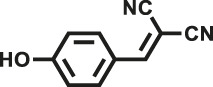	95	89
4	NO_2_	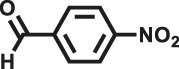	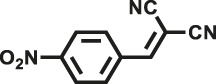	99	99
5	CN	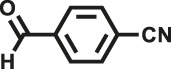	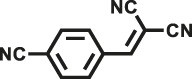	99	99
6	C(CH_3_)_3_	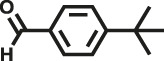	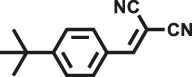	7	6
7	Ph	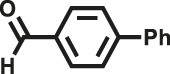	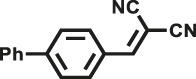	41	18
8	CH_3_	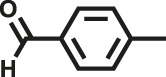	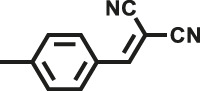	36	23
9	OCH_3_	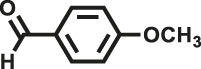	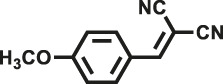	17	12

a Reaction conditions: A (1 mmol), B (1.1 mmol), TrPB-CMP or TePB-CMP (0.1 mmol), H_2_O (0.5 ml), toluene (1.5 ml). All reaction yields were obtained by the results of GC-MS.

To illustrate the high catalytic performance and gain further insights into the catalytic mechanism, additional comparison experiments were performed to evaluate the catalytic activity of pyrrole-based small molecules, i.e. TrPB and TePB (Scheme 1) and linear polypyrrole toward Knoevenagel condensation under the same conditions as those of CMPs. As shown in Supplementary Table S3, the catalytic activity of TrPB, TePB and polypyrrole was not obvious, which was similar to without any catalyst. Consequently, compared with non-porous analogues, the open porous structure allows the reactants to easily enter the catalytic center. In addition, the larger the specific surface area of the pore, the better the catalytic performance. Due to the high specific surface area and microporous character, benzimidazole-based CMPs (BPOP-1 and BPOP-2) was favorable for the accessibility of substrates to catalytic active sites inside the framework (Wang et al., 2015), which make the heteroatoms on the pore wall fully exhibit catalytic activity. Moreover, Similar pore restriction effects also appeared in other catalytic reactions (Mackintosh et al., 2008; Hu et al., 2020; Yang et al., 2020).

**FIGURE 3:**
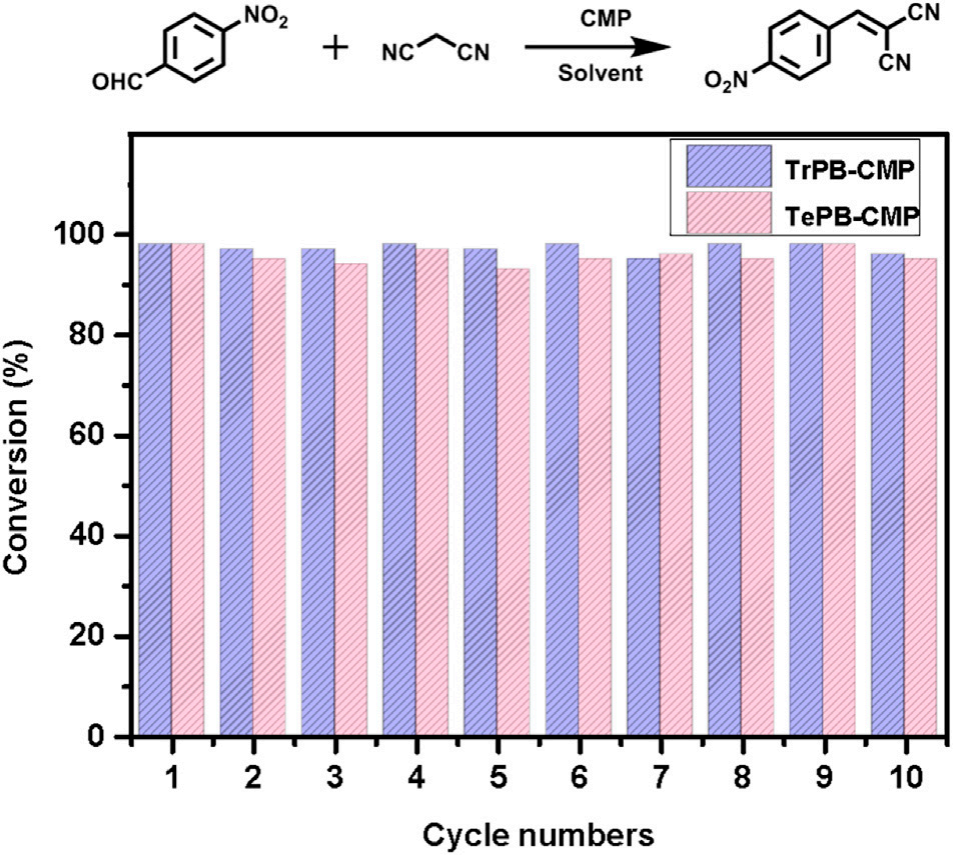
Recyclability of TrPB-CMP (blue) and TePB-CMP (red) as catalysts with *p*-nitrobenzaldehyde as the substrate.

As for testing the rates of the reactions, *p*-nitrobenzaldehyde was used as the substrate which catalyzed by both TrPB-CMP and TePB-CMP (Supplementary Figures S15, S16, ESI). The results suggested the substrates are quickly converted into the products within 30 min for both CMP-catalyzed reactions and reached the maximum conversion within 1 h. The conversion rate of TrPB-CMP is faster than that of TePB-CMP, which was probably benefited from the bigger pore size and specific surface area of TrPB-CMP. As to the recyclability of both CMP catalysts, as shown in Figure 3, the catalytic activities of both CMPs are basically unchanged within 10 cycles. After 10 cycles, FT-IR spectra of both recycled CMPs appeared the same as those of the pristine CMPs, which suggests the structures of both CMPs are robust and intact (Supplementary Figures S7, S8, ESI). In addition, after 10 cycles, the N_2_ adsorption tests indicated the BET specific surface areas of TrPB-CMP and TePB-CMP were 800 and 781 m^2^g^−1^, respectively, both of which were only slightly decreased compared with those of the pristine CMPs (Supplementary Figures S17, ESI). Consequently, it reveals both CMPs serve as efficient heterogeneous catalysts with excellent recyclability.

## Conclusion

In summary, two new pyrrole-based conjugated microporous polymers were successfully synthesized by self-polymerization of 1,3,5-tri-(pyrrol-2-ly)benzene or 1,2,4,5-tetra (pyrrol-2-ly)benzene. These two CMPs effectively catalyzed Knoevenagel condensation reaction with diverse substrates and showed excellent recycling performance, which was attributed to the open pore channels, large specific surface area and abundant heteroatoms as active sites within CMPs. This work suggests a new approach to fabricate pyrrole-based heterogenous catalysts. Additionally, both CMPs exhibit broad absorptions between 250 and 2400 nm, which might promise application potentials in photocatalysis.

## Data Availability

The original contributions presented in the study are included in the article/Supplementary Material, further inquiries can be directed to the corresponding authors.
