# Supplementary material for: Pyrrole-Based Conjugated Microporous Polymers as Efficient Heterogeneous Catalysts for Knoevenagel Condensation
Source: Front Chem. 2021 May 10;9:687183. doi: 10.3389/fchem.2021.687183 (PMC8141711; doi:10.3389/fchem.2021.687183)
Supplement: Supplementary file 1 [file DataSheet1.PDF]

Supplementary Material

Contents

Section 1. Materials and Methods

Section 2. Synthetic Procedures

Section 3. NMR Spectra

Section 4. FT-IR Spectra

Section 5.TEM Images of CMPs

Section 6. PXRD of CMPs

Section 7. Thermogravimetric Analysis

Section 8. UV-vis Absorption Spectra

Section 9. Solid-State ^{13}C CP/MAS NMR Spectra

Section 10. Knoevenagel Condensation Catalyzed by CMPs

Section 11. Reaction Rates of Knoevenagel Condensation as a Function of Time

Section 12. N_2 Sorption Isotherms

Section 13. Model of TrPB-CMP and TePB-CMP

Section 14. Catalytic mechanism

Section 1. Materials and Methods

The chemicals and solvents like 1-(tert-butoxycarbonyl)-pyrrole-2-boronic acid, 1,2,4,5-tetra-bromobenzene, 1,3,5-tris-bromobenzene, *N*,*N*-dimethylformamide, ethylene glycol, potassium carbonate, bis(triphenylphosphine)palladium(II) chloride, chloroform, ferric chloride, methanol, hydrochloric acid. All chemicals and solvents were used as received without further purification.

^1H NMR and ^{13}C NMR spectra of all organic compounds were recorded on a Bruker AVANCE III-400 NMR spectrometer. The mass spectra were recorded by matrix-assisted laser desorption ionization time-of-flight (MALDI-TOF) mass spectroscopy with Bruker Autoflex speed TOF mass spectrometer. High-resolution mass spectra were obtained with a TOF-QII mass spectrometer. The ^{13}C cross-polarization magic angle spinning (CP/MAS) spectra were recorded with a 4 mm double resonance MAS probe and at a MAS rate of 10.0 kHz with a contact time of 2 ms (ramp 100) and a pulse delay of 3 s. Fourier transform infrared (FT-IR) spectra were recorded in reflection mode on a Bruker Alpha spectrometer in the range of 400-4000 cm^{-1}. UV-vis spectra were measured on PerkinElmer Lambda 750 spectrometer, the sweep range was set from 250 to 2400 nm. The thermal properties of TrPB-CMP and TePB-CMP was evaluated using a thermogravimetric analysis (TGA) with a differential thermal analysis instrument (TA Instruments TGA Q50-1918 analyzer) over the temperature range from 35 to 800 °C in flowing N_2 with a heating rate of 10 °C min^{-1} using an empty Al_2O_3 crucible as the reference. Surface areas were measured by nitrogen adsorption and desorption at 77 K using a Bel Japan Inc. model BELSOPR-mini analyzer and the samples were degassed at 150 °C for 8 h under vacuum (10^{-5} bar) before analysis. The pore size distribution was calculated from the adsorption branch by nonlocal density functional theory (NLDFT) method. The powder X-ray diffraction (PXRD) patterns were recorded on X-ray diffractometer (RIGAKU SMARTLAB9KW). Transmission electron microscopies (TEM) and high-resolution transmission electron microscopies (HR-TEM) were performed on a FEI model Tecani 20 microscope and a JEOL model JEM-2100F. Field emission scanning electron microscopies (FE-SEM) were performed on a JEOL model JSM-6700 microscope operating at an accelerating voltage of 5.0 kV. The specific procedure of GC-MS was shown as follow: the initial temperature is 100 °C and keeping for 3 min, then warming up to 250 °C at a rate of 10 °C min^{-1} and keeping for another 3 min. The chromatographic column used in this method is Rtx-5MS, (solvent : acetone, solvent delay : 4 min). All products were identified by the comparison of GC retention time and mass spectra with those of the authentic samples. The yields for the catalytic reaction were calculated based on GC-MS (Shimadzu GCMS-QP2010 SE) data.

Section 2. Synthetic Procedures

Synthesis of 1,3,5-tri(pyrrol-2-ly)benzene (TrPB)

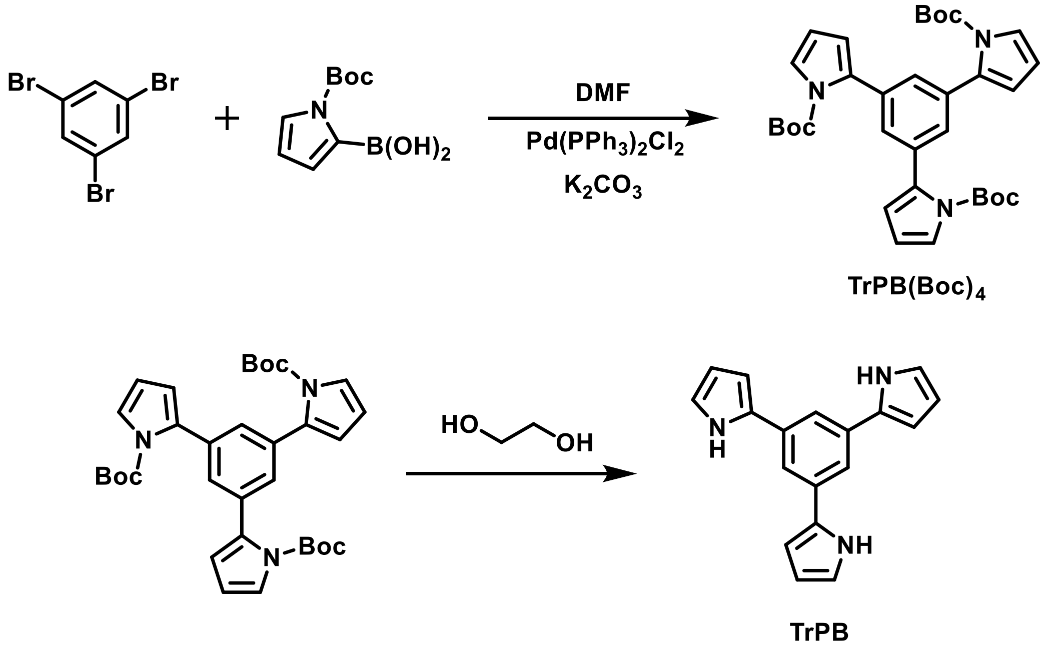

Scheme S2. Synthetic routes for 1,3,5-tri(pyrrol-2-ly)benzene (TrPB).

1,3,5-Trisbromobenzene (315 mg, 1 mmol), 1-(tert-butoxycarbonyl)-pyrrole-2-boronic acid (791 mg, 3.75 mmol), and $PdCl_2(PPh_3)_2$ (116 mg, 40 mmol%) were added to a mixture of 1.0 M K_2CO_3 aq. (5 mL) and DMF (25 mL). The reaction system was degassed by bubbling with N_2 for 5 min. The mixture was heated at 80 °C for 12 h. After cooling to room temperature, the mixture was extracted with EtOAc. The combined organic layer was washed with water and brine, and then dried over Na_2SO_4. After removal of solvent, the yellow oily liquid (TrPB(Boc)$_4$, 200 mg) was collected. Due to difficult separation, the product was used without further purification.

A suspended solution of TrPB(Boc)$_4$ (200 mg, 0.27 mmol) in ethylene glycol (30 mL) was bubbled with argon for 5 min, and then the mixture was heated at 160 °C for 1 h. After cooling to room temperature, water was added into the mixture. The solids were collected by filtration. The product was purified by silica gel chromatography (PE/EtOAc = 4/1) to obtain TrPB as a white solid (152 mg, yield: 56%). ^{1}H NMR (400 MHz, DMSO-d_6): δ (ppm) 11.27 (s, 3H), 7.71 (b, 3H), 6.90 (b, 3H), 6.63(b, 3H), 6.17 (b, 3H).^{13}C NMR (100 MHz, DMSO-d_6): δ (ppm) 133.63, 131.46, 119.20, 115.98, 109.04, 105.85. MALDI TOF-MS: m/z Calc. for $C_{18}H_{15}N_3$: 273.34 $[M]^+$, found 273.269.

Synthesis of 1,2,4,5-tetra(pyrrol-2-ly)benzene (TePB)[S1]

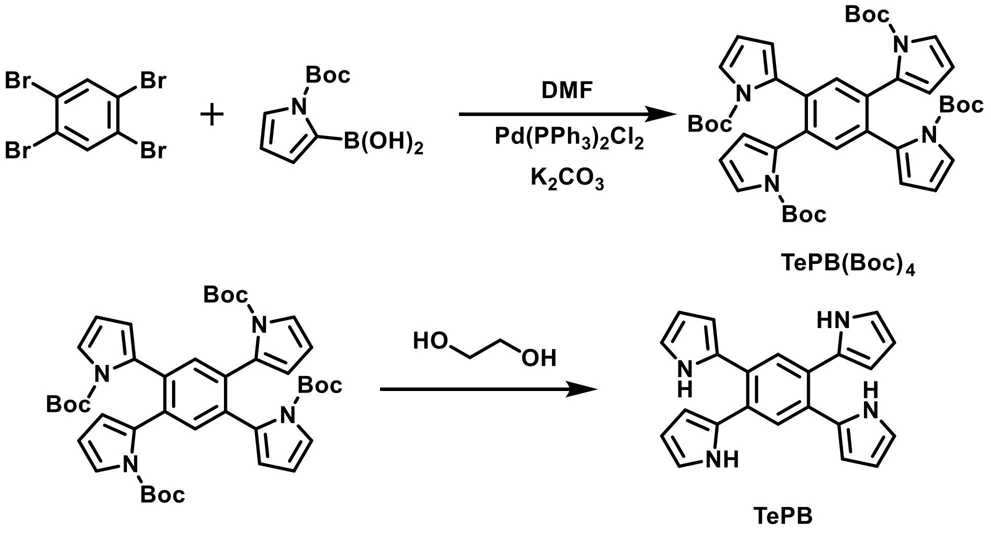

Scheme S1. Synthetic routes for 1,2,4,5-tetra(pyrrol-2-ly)benzene (TePB).

1,2,4,5-Tetrabromobenzene (311 mg, 0.80 mmol) and 1-(tert-butoxycarbonyl)-pyrrole-2-boronic acid (844 mg, 4.00 mmol) were added to a mixture of 1.0 M K_2CO_3 aq. (4 mL) and DMF (20 mL). Argon was flashed into the reaction mixture for 5 min to degas the remained oxygen and then $PdCl_2(PPh_3)_2$ (234 mg, 0.32 mmol, 40 mol%) was added to reaction mixture. The resulted reaction mixture was heated at 80 $^{\circ}$C for 12 h. After cooling to room temperature, the mixture was extracted with EtOAc. The combined organic layer was washed with water and brine, and then dried over Na_2SO_4. The crude was purified by silica gel column chromatography (PE/EtOAc = 10/1) and rinsed with hexane to give TePB(Boc)$_4$ as white powder in 40% yield (236 mg, 0.32 mmol). ^{1}H NMR (400 MHz, CD_2Cl_2): δ (ppm) 7.23 (s, 2H), 7.14 (m, 4H), 6.08 (m, 4H), 5.98 (m, 4H), 1.41 (s, 36H). ^{13}C NMR (100 MHz, CD_2Cl_2): δ (ppm) 148.97, 133.64, 133.24, 132.25, 121.89, 115.05, 110.30, 83.62, 27.94.

A solution of TePB(Boc)$_4$ (200 mg, 0.27 mmol) in ethylene glycol (30 mL) was degassed by bubbling with argon for 5 min, and then the mixture was heated at 160 $^{\circ}$C for 1 h. After cooling to room temperature, the mixture was extracted with CH_2Cl_2. The combined organic layer was washed with water and brine, and then dried over Na_2SO_4. After removal of solvent, the crude product was rinsed with hexane to give TePB as a pale-white solid in 90% yield (82 mg, 0.24 mmol). ^{1}H NMR (400 MHz, $CDCl_3$): δ (ppm) 8.00 (b, 4H), 7.61 (s, 2H), 6.73 (m, 4H), 6.40 (m, 4H), 6.26 (m, 4H). ^{13}C NMR

(100 MHz, $CDCl_3$): δ (ppm) 130.99, 130.62, 129.30, 119.14, 109.31, 108.40. MALDI TOF-MS: m/z Calc. for $C_{22}H_{18}N_4$: 338.41 $[M]^+$, found 338.234.

Synthesis of TrPB-CMP

1,3,5-Tri(pyrrol-2-ly)benzene (137 mg, 0.5 mmol) was dissolved 30 mL of chloroform and then transferred dropwise to a suspension of ferric chloride (477 mg, 3.75 mmol) in 20 mL of chloroform. The solution mixture was stirred at room temperature for 36 h under nitrogen protection, and then 100 mL of methanol was added to the above reaction mixture. The resulting mixture was kept stirring for another one hour and the precipitate was collected by filtration. After washed with methanol, the obtained solid was stirred vigorously in hydrochloric acid solution (37%) for 2 h. The suspension was then filtered and washed with water and methanol. The target polymer (TrPB-CMP) was collected (92 % in yield) and dried in vacuum oven at 110 °C overnight. FT-IR (ν, cm^{-1}): 3450, 1780, 1603, 1408, 1250, 1178, 1112, 1058, 789, 692, 534.

Synthesis of TePB-CMP

1,2,4,5-Tetra(pyrrole-2-yl)benzene (169 mg, 0.5 mmol) was dissolved 30 mL of chloroform and then transferred dropwise to a suspension of ferric chloride (477 mg, 3.75 mmol) in 20 mL of chloroform. The solution mixture was stirred at room temperature for 36 h under nitrogen protection, and then 100 mL of methanol was added to the above reaction mixture. The resulting mixture was kept stirring for another hour and the precipitate was collected by filtration. After washed with methanol, the obtained solid was stirred vigorously in hydrochloric acid solution (37%) for 2 h. The suspension was then filtered and washed with water and methanol. The desired polymer (TePB-CMP) was collected (90 % in yield) and dried in vacuum oven at 110 °C overnight. FT-IR (ν, cm^{-1}): 3450, 1758, 1712, 1614, 1408, 1250, 1178, 1046, 766, 523.

Table S1. Elemental analysis of TrPB-CMP and TePB-CMP.

TrPB-CMP	C%	N%	H%

	**Cal.**	80.0	15.5	5.6
	**Exp.**	80.5	12.1	7.5
TePB-CMP		C%	N%	H%
	**Cal.**	79.0	16.8	5.4
	**Exp.**	79.2	13.8	7.0

Section 3. NMR Spectra.

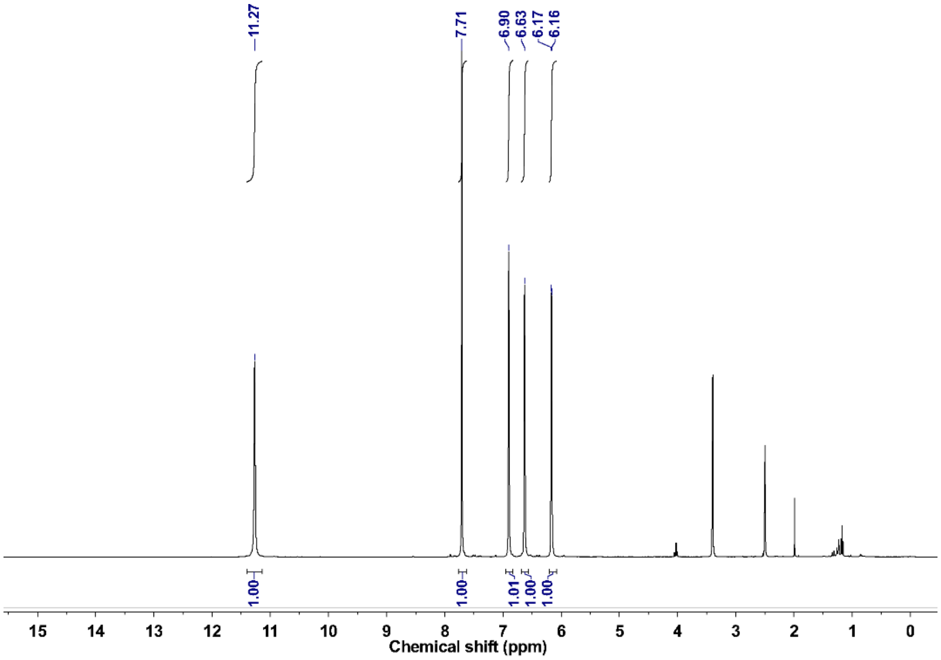

Figure S1. ^{1}H NMR spectra of TrPB in DMSO-d_6.

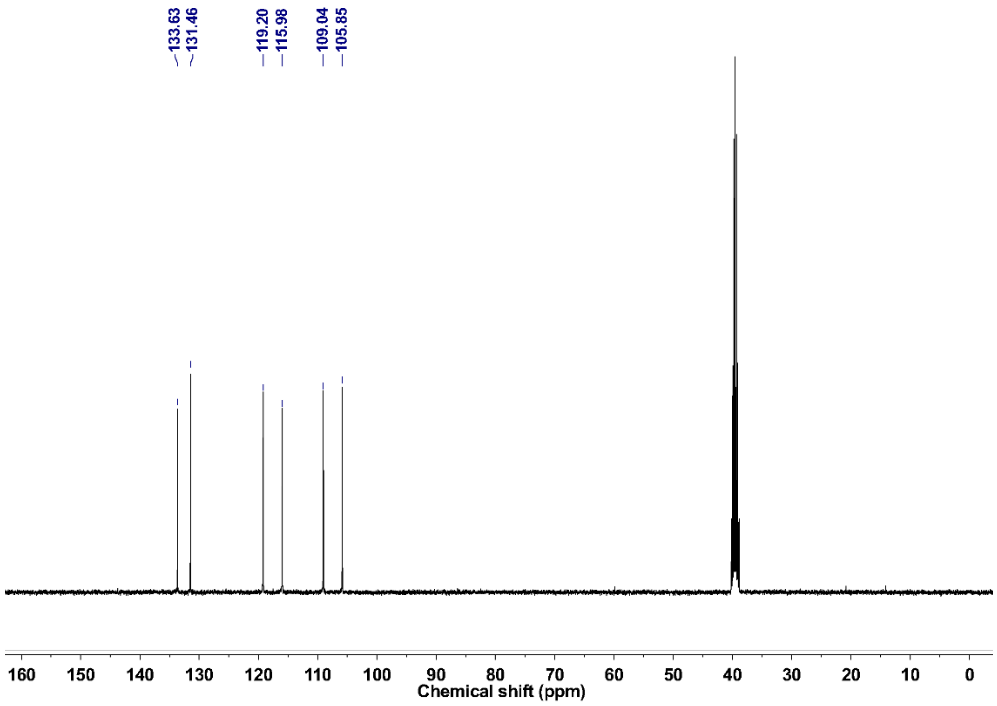

Figure S2. ^{13}C NMR spectra of TrPB in DMSO-d_6.

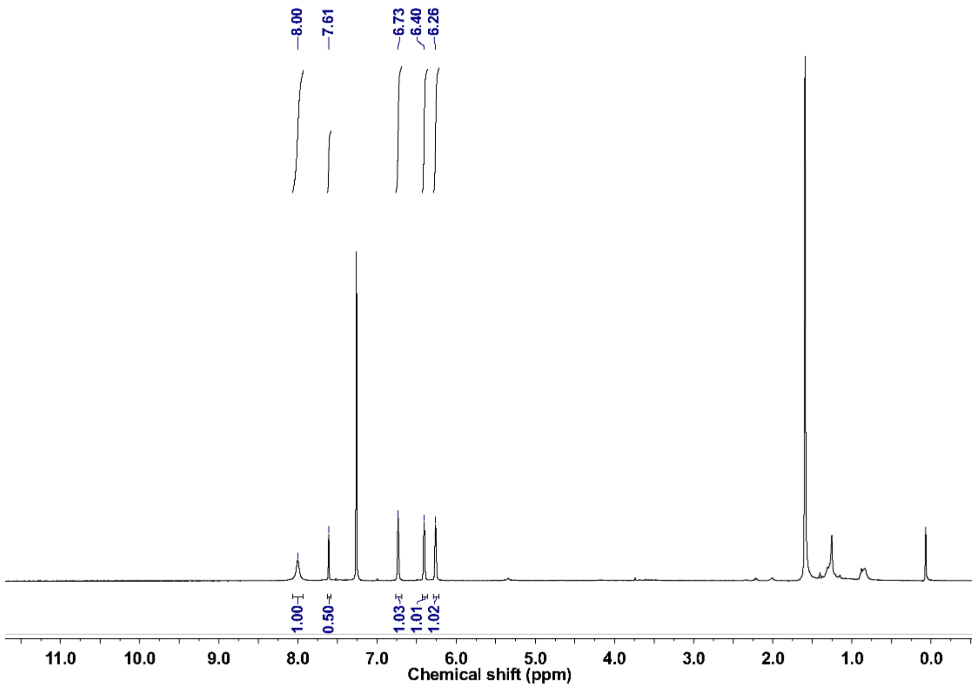

Figure S3. ^{1}H NMR spectra of TePB in $CDCl_3$.

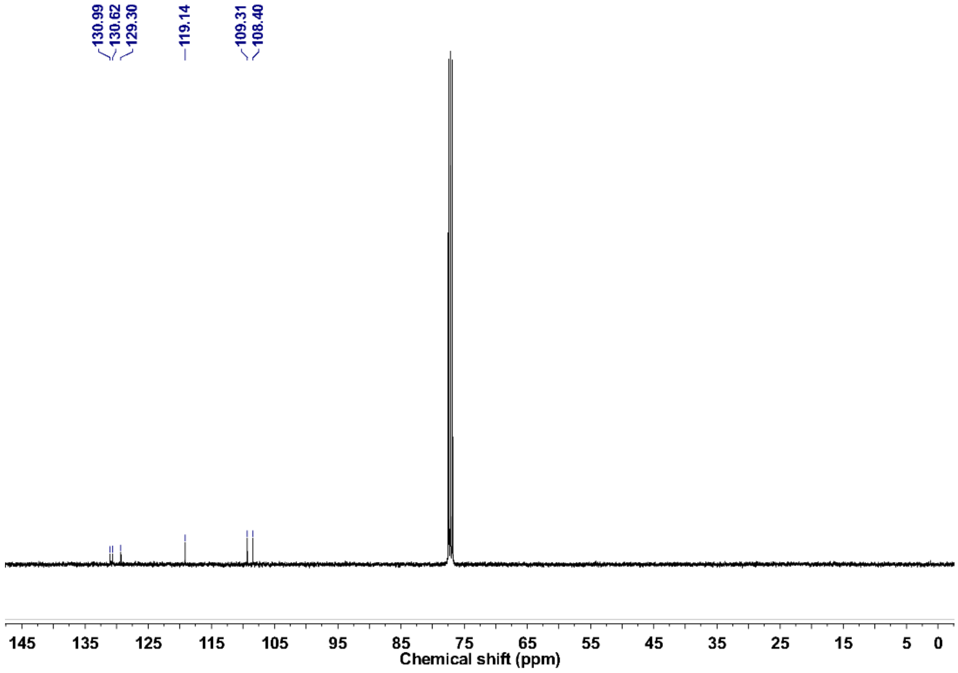

Figure S4. ^{13}C NMR spectra of TePB in $CDCl_3$.

Section 4. FT-IR Spectra

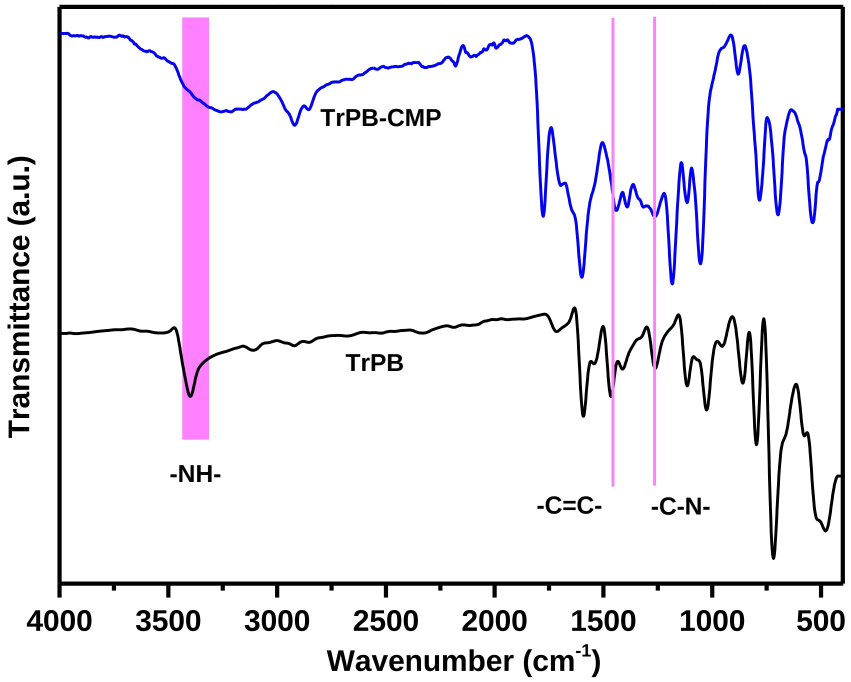

Figure S5. FT-IR spectra comparison of TrPB and TrPB-CMP.

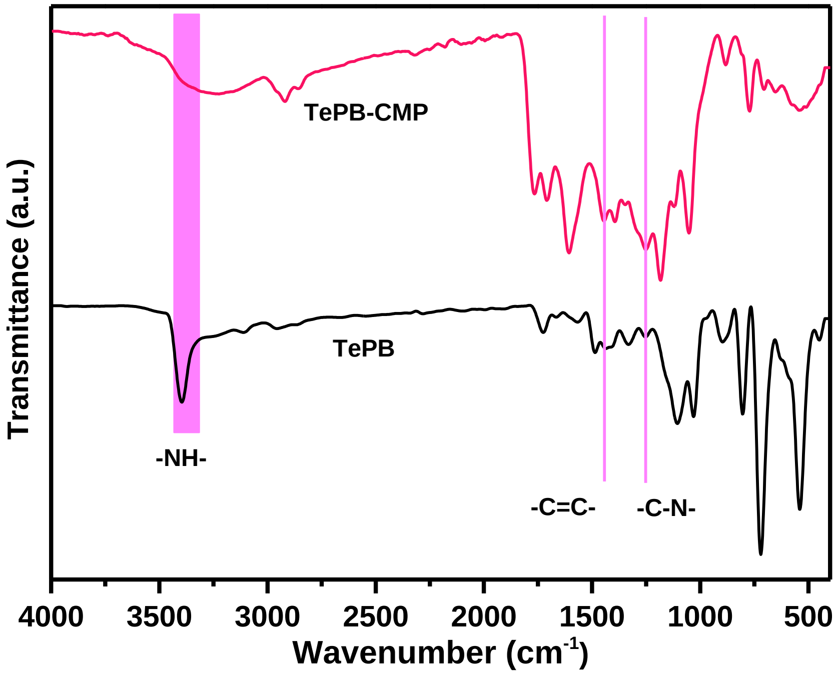

Figure S6. FT-IR spectra comparison of TePB and TePB-CMP.

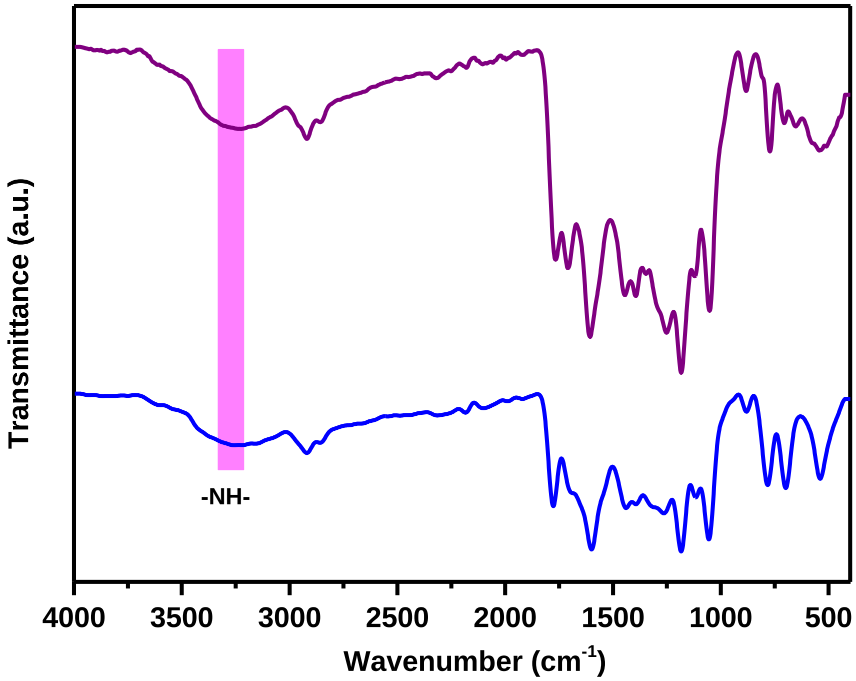

Figure S7. FT-IR spectra of TrPB-CMP after ten cycles of catalysis (blue line: before catalytic cycle, purple line: after catalytic cycle).

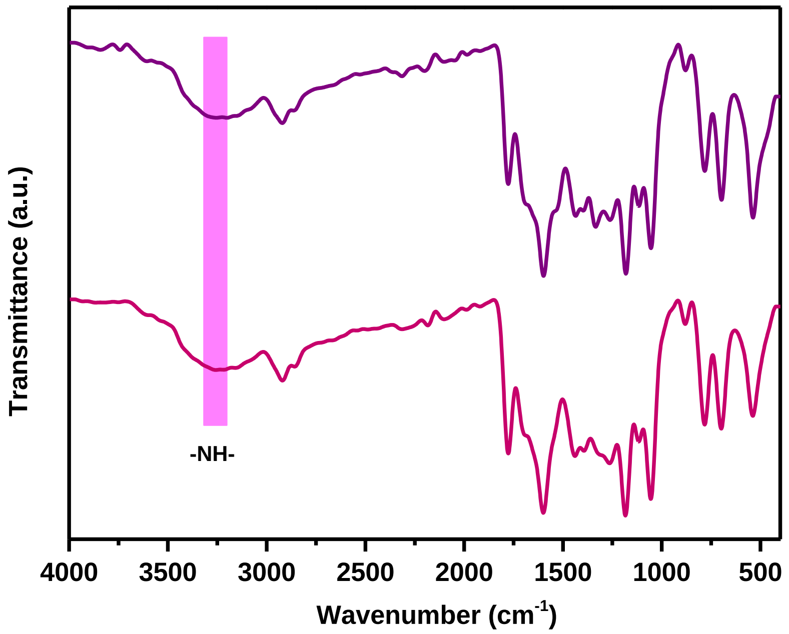

Figure S8. FT-IR spectra of TePB-CMP after 10 cycles of catalysis (red line: before catalytic cycle, purple line: after catalytic cycle).

Section 5. TEM Images of CMPs

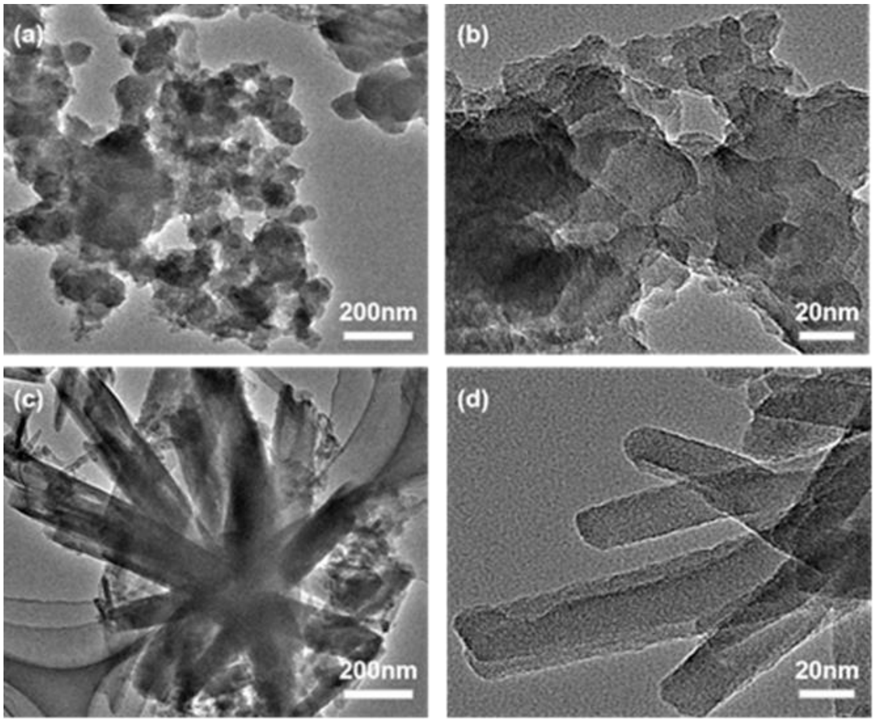

Figure S9. TEM images of (a, b) TrPB-CMP and (c, d) TePB-CMP.

Section 6. PXRD of CMPs

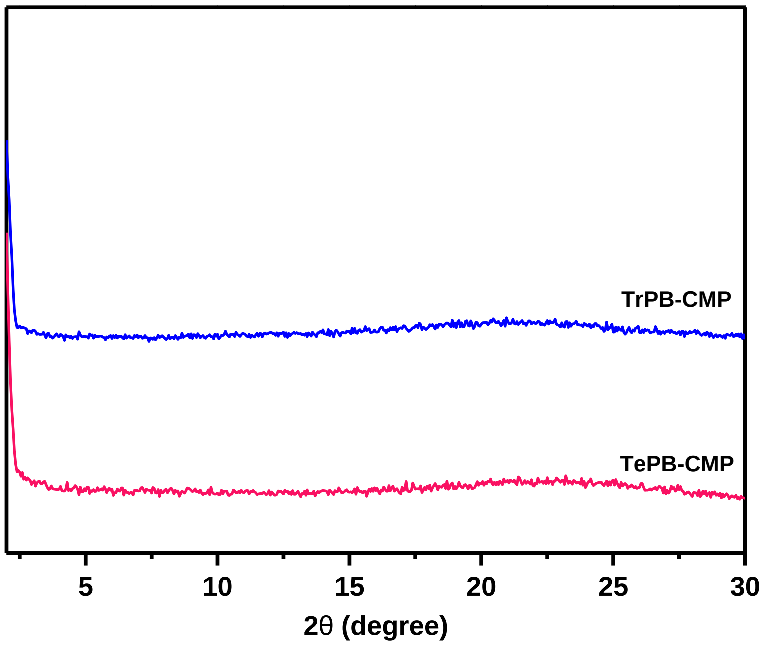

Figure S10. Powder X-ray diffraction of CMPs (blue: TrPB-CMP, red: TePB-CMP).

Section 7. Thermogravimetric Analysis

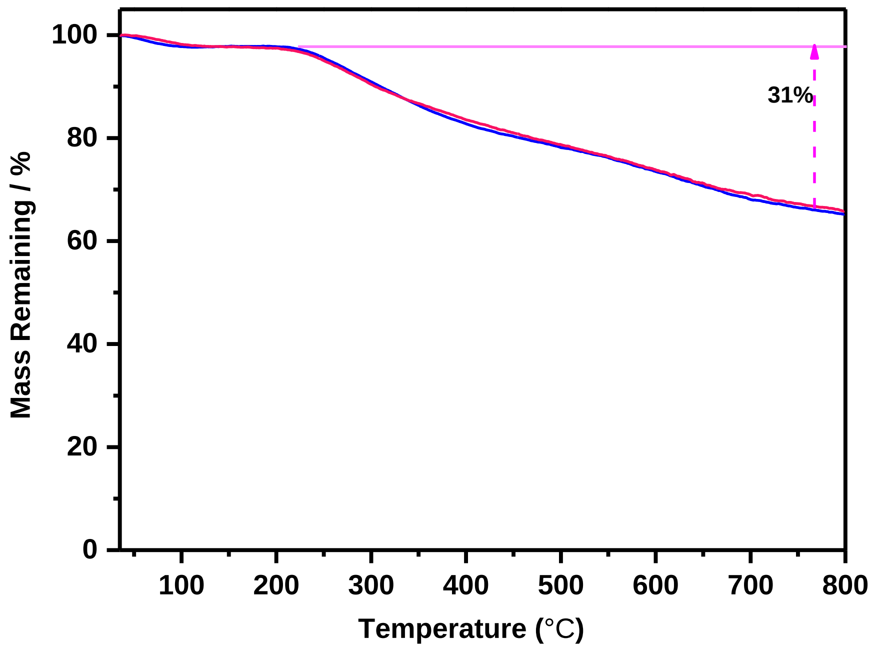

Figure S11. Thermogravimetric analysis for CMPs (blue: TrPB-CMP, red: TePB-CMP).

Section 8. UV-vis Absorption Spectra

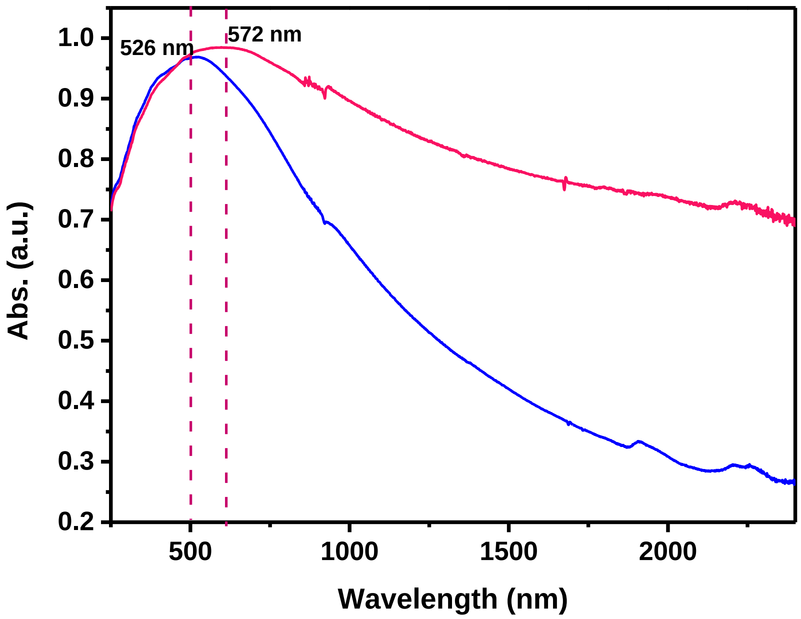

Figure S12. UV-vis absorption spectra of CMPs (blue: TrPB-CMP, red: TePB-CMP).

Section 9. Solid State ^{13}C CP/MAS NMR Spectra

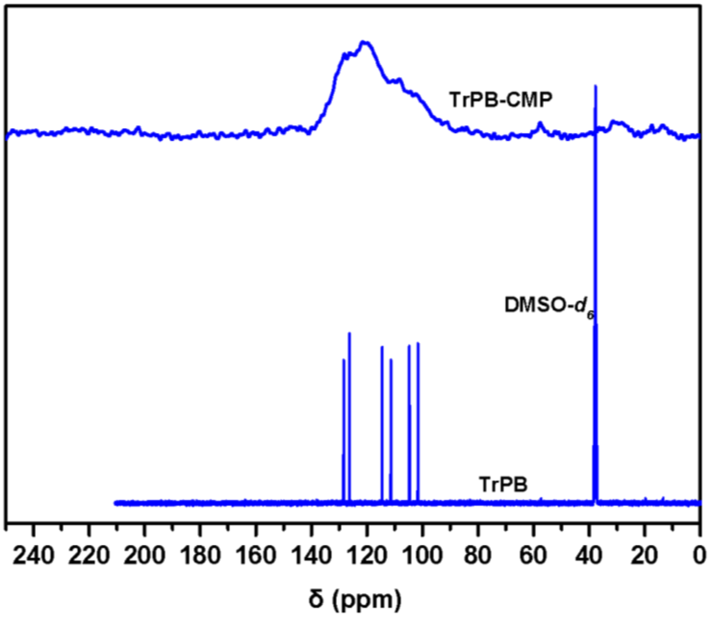

Figure S13. Solid-state ^{13}C CP-MAS NMR spectra of TrPB-CMP compared to the ^{13}C NMR spectrum of TrPB in DMSO-d_6.

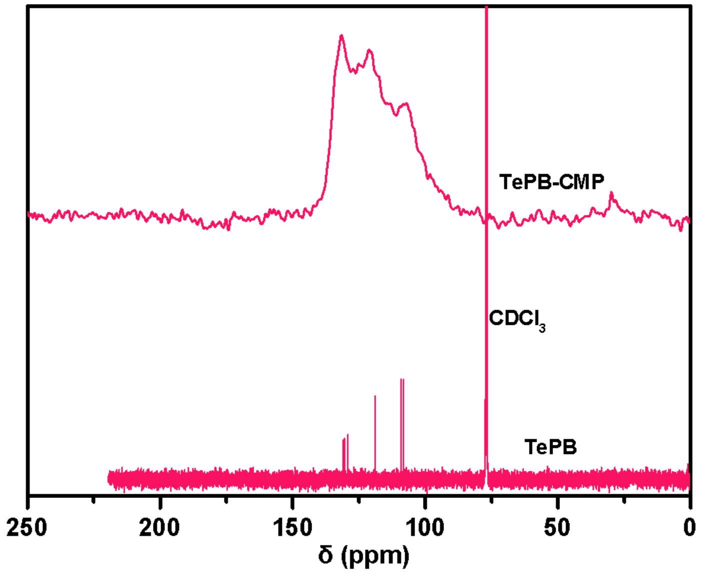

Figure S14. Solid-state ^{13}C CP-MAS NMR spectra of TePB-CMP compared to the ^{13}C NMR spectrum of TePB in $CDCl_3$.

Section10. Knoevenagel Condensation Catalyzed by CMPs

Table S2. Optimization of Knoevenagel condensations of *p*-nitrobenzaldehyde with malononitrile catalyzed by TrPB-CMP or TePB-CMP[a].

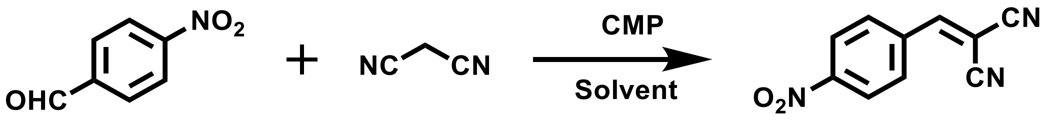

Entry	Toluene (mL)	Water (mL)	Temp. (°C)	Time (h)	Yield (%)	
					TrPB-CMP	TePB-CMP
1	1.5	0.5	80	1	99	99
2	2	0	60	1	22	13
3	1.5	0.5	60	1	>99	>99
4	1	1	60	1	>99	>99
5	1.5	0.5	40	1	63	47

[a]Reaction conditions: TrPB-CMP (41 mg) or TePB-CMP (51 mg), *p*-nitrobenzaldehyde (1 mmol), malononitrile (1.1 mmol). All yields determined by GC-MS analysis.

Table S3. Knoevenagel condensations of *p*-nitrobenzaldehyde with malononitrile catalyzed by the corresponding monomers and polypyrrole [a].

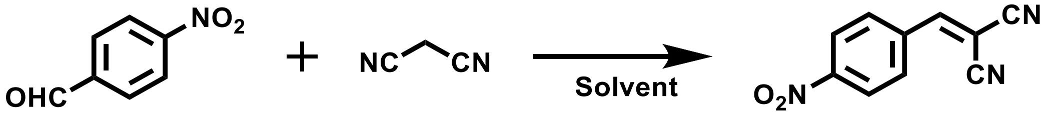

Entry	Toluene	Water	Temp. (°C)	Catalyst	Time (h)	Yield (%)
1	1.5 mL	0.5 mL	60	-	1	44
2	1.5 mL	0.5 mL	60	TrPB	1	51
3	1.5 mL	0.5 mL	60	TePB	1	55
4	1.5 mL	0.5 mL	60	Polypyrrole	1	48

[a]Reaction conditions: All yields determined by GC-MS analysis.

Table S4. Performance comparison on Knoevenagel condensations of *p*-nitrobenzaldehyde with malononitrile catalyzed by reported organic porous polymers.

Entry	Catalyst	Solvent	Temp. (°C)	Time (h)	Yield (%)	BET ($m^2 g^{-1}$)	Ref.
1	HCP-TCPP	Dioxane/H_2O	RT	3	92	1050	S2
2	HP_E-CMP	Dioxane/H_2O	RT	3	99	662	S3
3	MOF 1·Cd	Malononitrile	60	1	99	-	S4
4	MMPF-Mn	Toluene	100	2.5	98	-	S5
5	Fe-POP-1	Water/EtOH	RT	4	98	875	S6
6	A_2B_2-Por-COF	Water/Toluene	60	1	99	730	S7
7	TrPB-CMP	Water/Toluene	60	1	99	810	This work
8	TePB-CMP	Water/Toluene	60	1	99	800	This work

Section 11. Reaction Rates of Knoevenagel condensation as a Function of Time

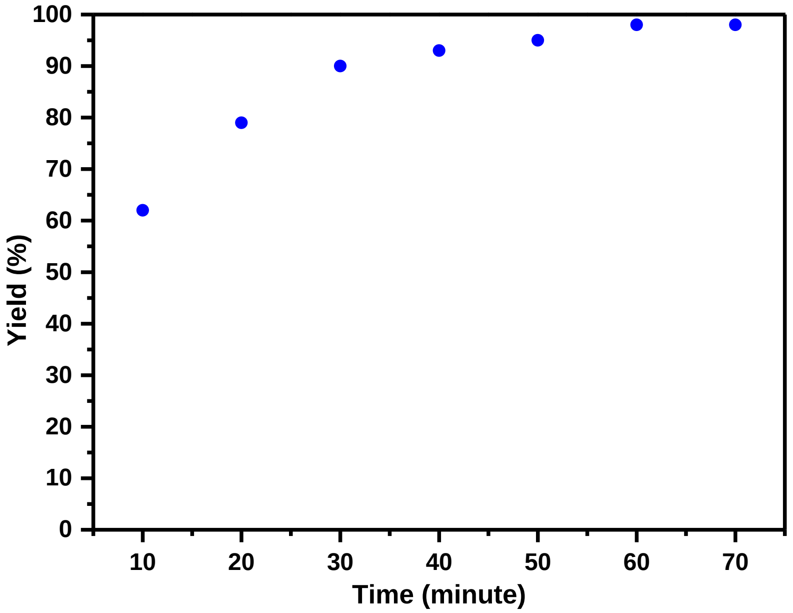

Figure S15. Yield of 2-(4-Nitrobenzylidene)malononitrile as a function of time in the Knoevenagel condensation reactionwith *p*-nitrobenzaldehyde as the substrate, catalyzed by TrPB-CMP.

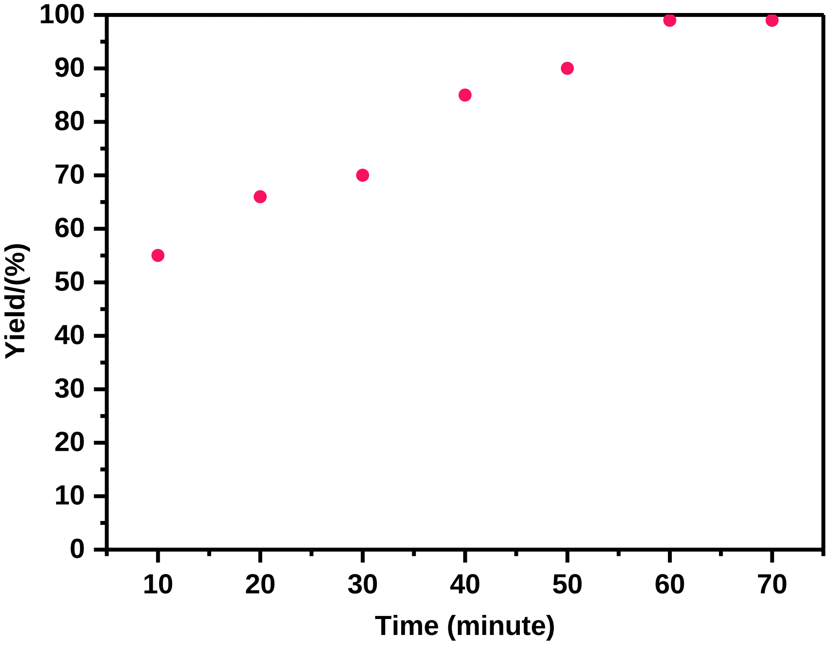

Figure S16. Yield of 2-(4-Nitrobenzylidene)malononitrile as a function of time in the knoevenagel condensation reaction with *p*-nitrobenzaldehyde as the substrate, catalyzed by TePB-CMP.

Section 12. N_2 Sorption Isotherms

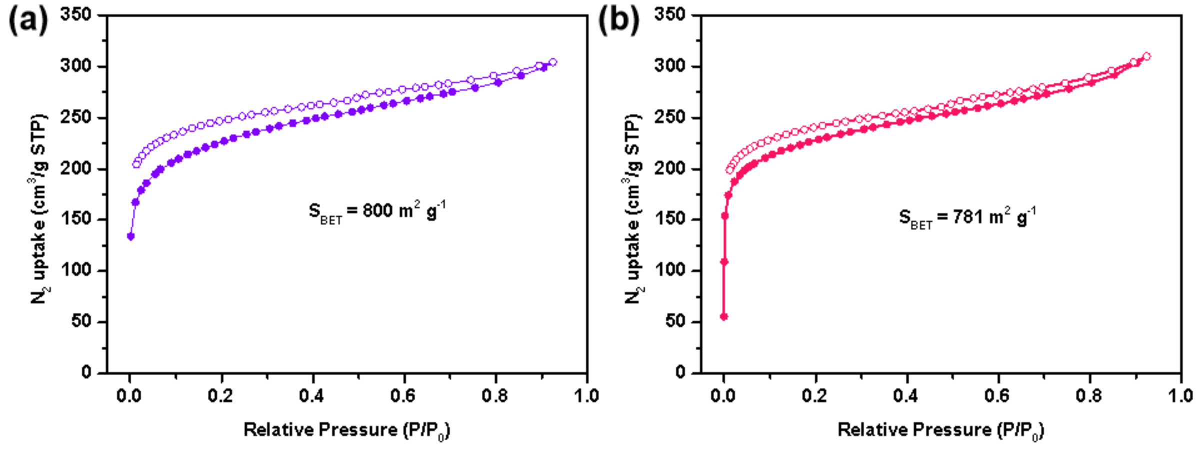

Figure S17. Nitrogen adsorption and desorption isotherms of (a) TrPB-CMP and (b) TePB-CMP at 77 K after ten cycles of catalysis.

Section 13. Model of TrPB-CMP

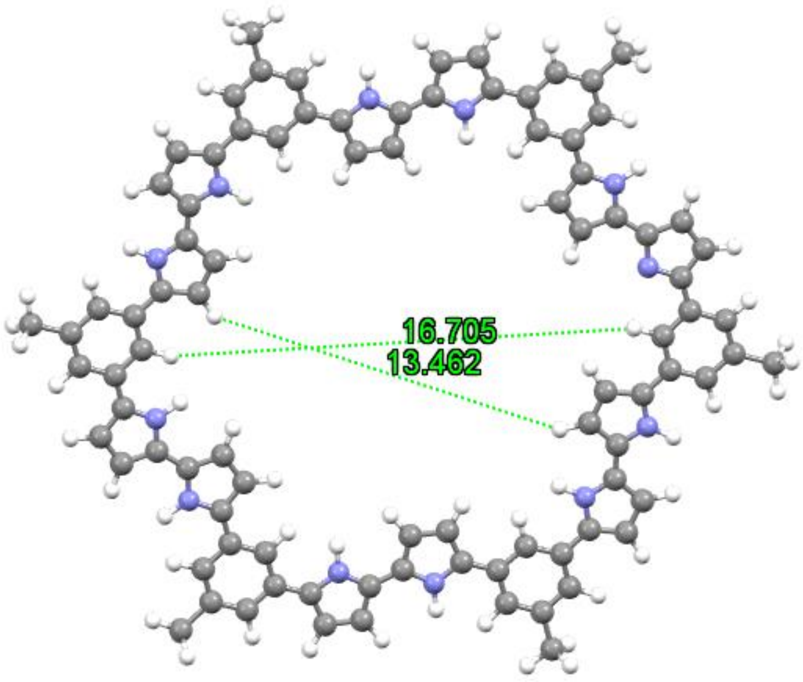

Figure S18. A typical segment of TrPB-CMP modeled by MM2 method.

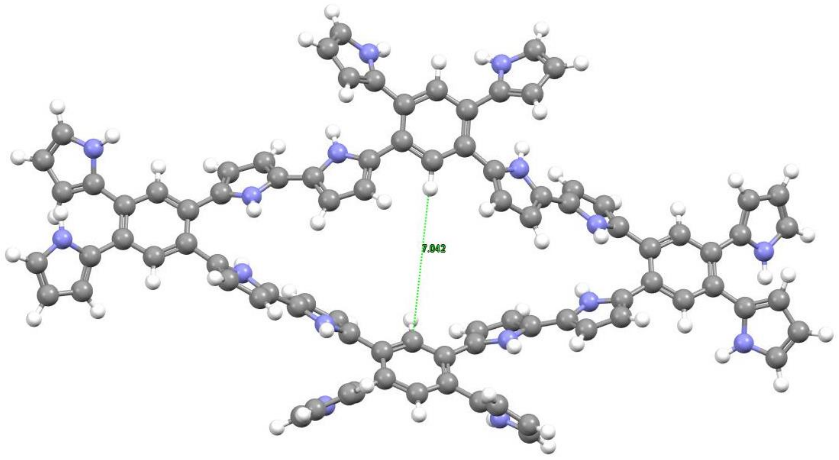

Figure S19. A typical segment of TePB-CMP modeled by MM2 method.

Section 14. Catalytic mechanism

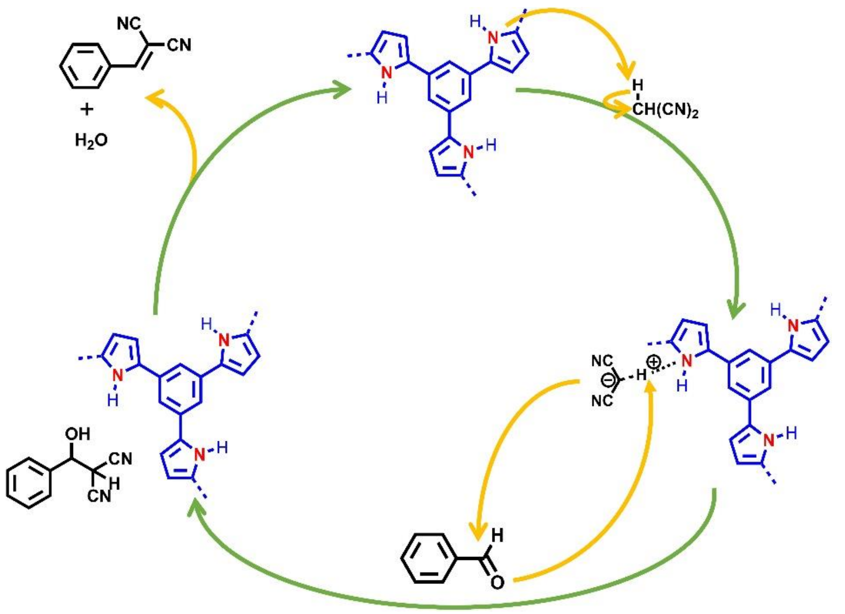

Figure S20. Proposed reaction mechanism for Knoevenagel condensation by catalyst CMP.

References

(S1) Xue, S., Kuzuhara, D., Aratani, N., and Yamada, H. (2019). Synthesis of a porphyrin(2.1.2.1) nanobelt and its ability to bind fullerene. *Org. Lett.* 21 (7), 2069-2072. doi: 10.1021/acs.orglett.9b00329

(S2) Feng, L. J., Wang, M., Sun, Z. Y., Hu, Y., and Deng, Z. T. (2016). Hypercrosslinked porous polyporphyrin by metal-free protocol: characterization, uptake performance, and heterogeneous catalysis. Designed Monomers and Polymers 20, 344-350. doi: 10.1080/15685551.2016.1259831

(S3) Liu, X., A, S., Zhang, Y., Luo, X., Xia, H., Li, H., et al. (2014). A porphyrin-linked conjugated microporous polymer with selective carbon dioxide adsorption and heterogeneous organocatalytic performances. *RSC Adv*. 4 (13), 6447-6453. doi: 10.1039/C3RA46988C

(S4) Jiang, W., Yang, J., Liu, Y. Y., Song, S. Y., and Ma, J. F. (2017). A stable porphyrin-based porous mog metal-organic framework as an efficient solvent-free catalyst for C-C bond formation. *Inorg. Chem.* 56 (5), 3036-3043. doi: 10.1021/acs.inorgchem.6b03174

(S5) Zhang, W., Jiang, P., Wang, Y., Zhang, J., and Zhang, P. (2015). Synthesis of two metal-porphyrin frameworks assembled from porphyrin building motifs, 5, 10, 15, 20-tetrapyridylporphyrin and their base catalyzed property. *Inorg. Chem Commun.* 61, 100-104. doi: 10.1016/j.inoche.2015.09.002

(S6) Modak, A., Mondal, J., and Bhaumik, A. (2013). Porphyrin based porous organic polymer as bi-functional catalyst for selective oxidation and knoevenagel condensation reactions. *Appl. Catal. A: Gen. 459*, 41-51. doi: 10.1016/j.apcata.2013.03.036

(S7) Hao, W., Chen, D., Li, Y., Yang, Z., Xing, G., Li, J., et al. (2019). Facile synthesis of porphyrin based covalent organic frameworks via an A_2B_2 monomer for highly efficient heterogeneous catalysis. *Chem. Mater.* 31 (19), 8100-8105. doi: 10.1021/acs.chemmater.9b02718
